# Supplementary material for: Mutated TP53 in Circulating Tumor DNA as a Risk Level Biomarker in Head and Neck Squamous Cell Carcinoma Patients
Source: Biomolecules. 2023 Sep 20;13(9):1418. doi: 10.3390/biom13091418 (PMC10527516; doi:10.3390/biom13091418)
Supplement: Supplementary file 1 [file biomolecules-13-01418-s001.zip › biomolecules-2496970-supplementary.pdf]

**Supplementary**

Table S1. **List of primers used for TP53 amplification and M13/P1 primers**

| **Primer Name** | **Sequence** |
|---|---|
| TP53ex2_F | GATCCCCACTTTTCCTCTTG |
| TP53ex2_R | CTTCCCACAGGTCTCTGCTA |
| TP53ex3_F | ACTGACTTTCTGCTCTTGTCTT |
| TP53ex3_R | GGACTGTAGATGGGTGAAAA |
| TP53ex4a_F | TGCTCTTTTCACCCATCTAC |
| TP53ex4a_R | AGGGACAGAAGATGACAGG |
| TP53ex4b_F | CACCAGCAGCTCCTACAC |
| TP53ex4b_R | GCATTGAAGTCTCATGGAAG |
| TP53ex5start_F | AGTTGCTTTATCTGTTCACTTGT |
| TP53ex5start_R | GTGCTGTGACTGCTTGTAGAT |
| TP53ex6_F | GATTCCTCACTGATTGCTCTT |
| TP53ex6_R | CACTGACAACCACCCTTAAC |
| TP53ex9_F | TTATGCCTCAGATTCACTTTTAT |
| TP53ex9_R | TTTGAGTGTTAGACTGGAAACTT |
| TP53ex10_F | ATATACTTACTTCTCCCCCTCCT |
| TP53ex10_R | GATGAGAATGGAATCCTATGG |
| TP53ex11_F | CACTCATGTGATGTCATCTCTC |
| TP53ex11_R | GGGAACAAGAAGTGGAGAA |
| TP53ex5_F | CAGCTGTGGGTTGATTCC |
| TP53ex5_R | AGACCTAAGAGCAATCAGTGAG |
| TP53ex7_F | ATCTTGGGCCTGTGTTATC |
| TP53ex7_R | GGTCAGAGGCAAGCAGAG |
| TP53ex8_F | TTGCTTCTCTTTTCCTATCCT |
| TP53ex8_R | ATAACTGCACCCTTGGTCTC |
| M13 | TGTAAAACGACGGCCAGT |
| P1 | CCTCTCTATGGGCAGTCG |

Table S2. **List of primers designed to target patient specific TP53 mutation**

| Primer Name | Sequence |
| --- | --- |
| TP53_266to82_F | CCTGAGTAGTGGTAATCTACTGG |
| TP53_266to82_R | GAGATTCTCTTCCTCTGTGC |
| TP53209_13_F | AGTTGCAAACCAGACCTCAG |
| TP53209_13_R | GAAATTTGCGTGTGGAGTAT |
| TP53_ex8end_F | CGCTTCTTGTCCTGCTTG |
| TP53_ex8end_R | GAGAATCTCCGCAAGAAAG |
| TP53_R175H_F | GCCATCTACAAGCAGTCACA |
| TP53_R175H_R | CTGCTCACCATCGCTATCTG |
| TP53ex5spl4_F | GACTTTCAACTCTGTCTCCTTC |
| TP53ex5a_R | TGTGGAATCAACCCACAG |
| TP53_242to41F | ACAACTACATGTGTAACAGTTCC |
| TP53_242to41R | CCAGTGTGATGATGGTGAGG |
| TP53_158_F | GTGCTGTGACTGCTTGTAGAT |
| TP53_158_R | CAGCTGTGGGTTGATTCC |
| TP53_35_F | CTCTTTTCACCCATCTACAGTC |
| TP53_35_R | ACAGCATCAAATCATCCATT |
| TP53_ex8end_F | CGCTTCTTGTCCTGCTTG |
| TP53_ex8end_R | GAGAATCTCCGCAAGAAAG |
| TP53_195_F | ATTCCTCACTGATTGCTCTTAG |
| TP53_195_R | AAGTGTTTCTGTCATCCAAATAC |
| TP53_242F | ACAACTACATGTGTAACAGTTCCT |
| TP53_257R | CTCCTGACCTGGAGTCTTC |
| TP53_224F | ACTTTTCGACATAGTGTGGTG |
| TP53_224R | CTGACAACCACCCTTAACC |
| TP53_237_F | CTGGAGTCTTCCAGTGTGAT |
| TP53_237_R | GTACCACCATCCACTACAACT |

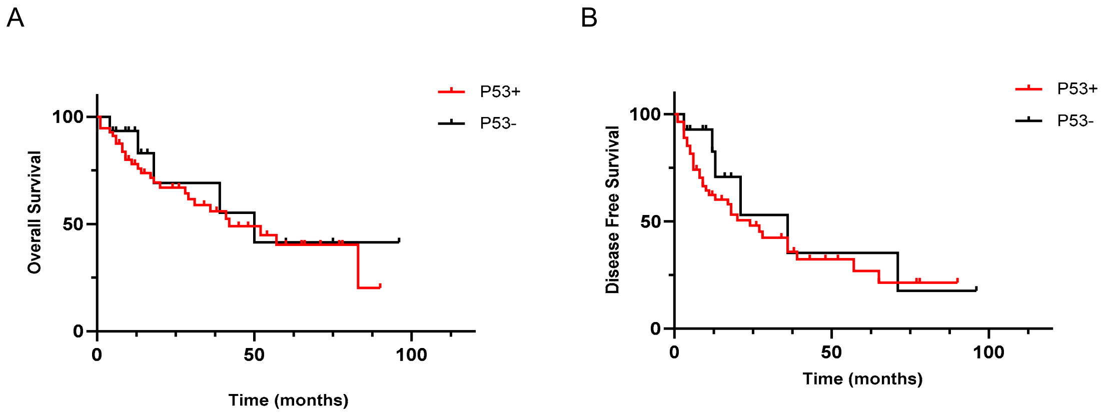


**Figure S1. Survival outcomes stratified by the presence of TP53 mutation in tumor DNA**. Kaplan Meier survival estimates of overall survival (A) and disease free survival (B) of the entire cohort (N=70) demonstrating no difference in clinical outcomes based on the presence of TP53 mutations.
